# Mentors without Borders

**DOI:** 10.1002/mgg3.246

**Published:** 2016-09-01

**Authors:** Maximilian Muenke

**Affiliations:** ^1^ Medical Genetics BranchNational Human Genome Research InstituteNational Institutes of HealthBethesdaMaryland20814

## Abstract

Mentors without Borders is a proposed international mentoring network that allows trainee geneticists to identify mentors from a list of volunteers who are not at one's own institution. It is an experiment, a matchmaker between a junior and a senior professional. These mentors do not replace the mentors at the home institution but allow the mentee, if desired, to identify mentors outside of their own institution. We envision that different ways of communicating and/or different mentor‐mentee relationships may prove beneficial to the trainee and the mentor.

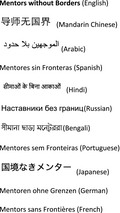

There is a special place in hell for women who do not help other women. *—* Madeleine Albright

In order to be a mentor, and an effective one, one must care. You must care. You don't have to know how many square miles are in Idaho, you don't need to know what is the chemical makeup of chemistry, or of blood or water. Know what you know and care about the person, care about what you know and care about the person you're sharing with. *—* Maya Angelou

Colleagues are a wonderful thing – but mentors, that's where the real work gets done. *—* Junot Diaz

## How it all Started: ICHG 2016 Meeting in Kyoto

In April 2016, at the 13th International Congress of Human Genetics (ICHG) in Kyoto, Japan, a small group of senior colleagues met with trainees from around the world for two career development sessions. Participants were at different stages of their careers and included graduate and medical students, postdoctoral trainees and medical residents, and junior faculty. Discussion topics ranged from “How to write a good manuscript” and “How to develop a successful academic career” to more individual questions such as “Where do I submit my manuscript?” and “Do I extend my fellowship to generate more research data?”. Afterward several colleagues from different continents met to informally become the founding members of a group that we called *Mentors without Borders*. The founding members were as follows: Judith Hall, University of British Columbia, Vancouver, Canada; Kenjiro Kosaki, Keio University, Tokyo, Japan; Yoichi Matsubara, National Research Institute for Child Health and Development, Tokyo, Japan; Naomichi Matsumoto, Yokohama City University Graduate School of Medicine, Yokohama, Japan; Max Muenke, National Institutes of Health, Bethesda, Maryland, USA; Giovanni Neri, Catholic University, Rome, Italy.

## Mentors Without Borders/Doctors Without Borders

**Figure 1:**
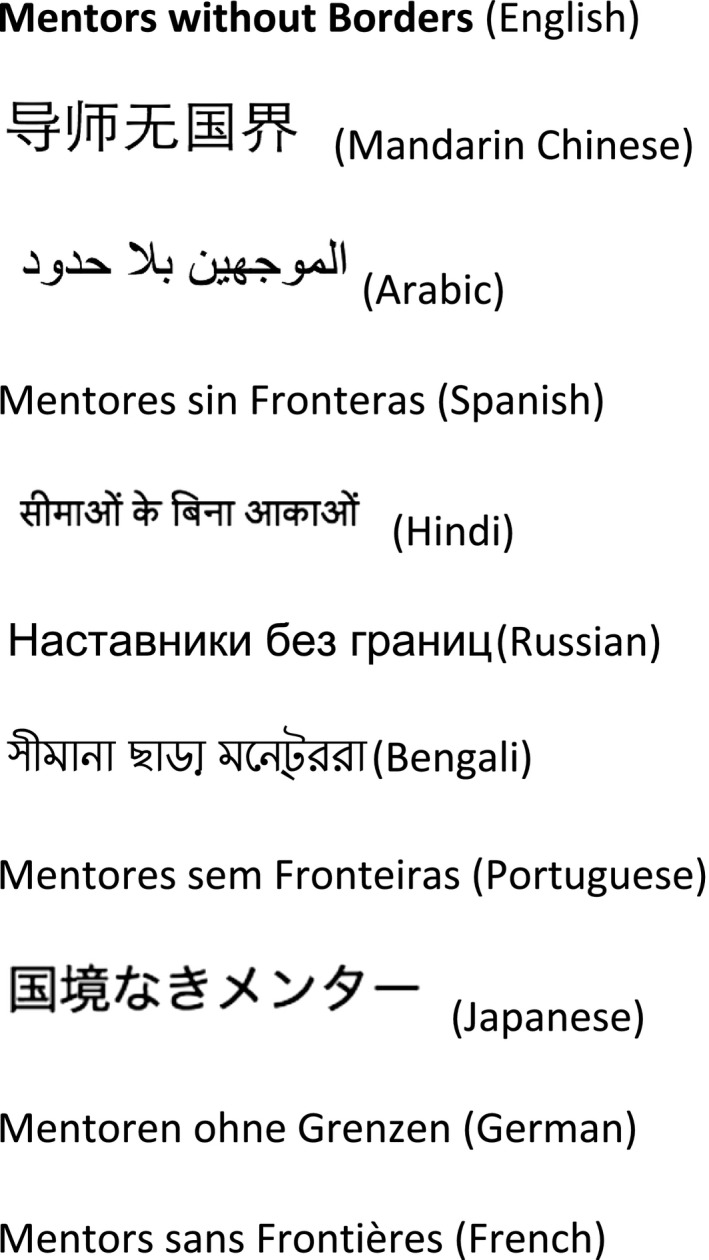
*Mentors without Borders* in the most common languages in the world.

The name *Mentors without Borders* (Fig. 1) is inspired by the original *Médecins Sans Frontières* (MSF) or *Doctors without Borders*. French physicians founded MSF in 1971 in the aftermath of the Nigerian Civil War (1967–1970) that led to the blockade and starvation of those who lived in the newly independent Biafra. MSF is an international, nongovernmental organization that delivers “emergency aid to people affected by armed conflict, healthcare exclusion and natural or man‐made disasters” (www.msf.org).

Many other professions have used the “… without Borders” name including: Accountants, Acupuncturists, Architects, Artists, Astronomers, Bankers, Builders, Chemists, Designers, Engineers, Entrepreneurs, Ergonomists, Executives, Farmers, Geoscientists, Homeopaths, Inventors, Lawyers, Librarians, MBAs, Mediators, Naturopaths, Nurses, Reporters, Researchers, Scientists, Sociologists, Teachers, Translators, Veterinarians, and even Magicians and Clowns (Schwietert 2010).

## Definition of Mentor

The word “mentor” originally comes from ancient Greek literature. In Homer's *Odyssey*, during his two decades' absence from home, Odysseus entrusted the upbringing and education of his son Telemachus to a loyal friend by the name of Mentor. Interestingly, at times the goddess Athena took the shape of Mentor, thus imparting a spiritual element to the Mentor character. The first known use of the Greek word *mentor* in Latin was in 1616 (Merriam‐Webster).

Today, definitions of “mentor” (the word can be used as a noun or a verb) include: an experienced and trusted advisor (Google); a trusted counselor or guide; a tutor, a coach (Merriam‐Webster); someone who teaches or gives help and advice to a less experienced and often younger person (Merriam‐Webster's Learner's Dictionary); a wise and trusted counselor or teacher; an influential senior sponsor (www.dictionary.com); an experienced and trusted person who gives another person advice over a period of time (Cambridge Dictionary).

Even though “mentoring” and mentoring relationships in different settings have been described for millennia, formal research on this topic has been published since the early 1980s including the seminal work by Kathy E. Kram entitled *Mentoring at Work: Developmental Relationships on Organizational Life* (1985). Searching “mentor”, “mentee”, and “mentoring” in PubMed (August 1, 2016) resulted in retrieval over 10,000 articles, many of them in the nursing literature (Chen et al. 2016; Gruber‐Page 2016; Sood et al. 2016) and in articles of higher education (Fleming et al. 2013) including physicians during residency training (Fig. 1).

## My Personal Experience

Over the years I have sought out and have had many mentors. Growing up in rural northern Germany in a resource‐poor family, I remember fondly the impact of teachers and how they mentored me: a piano teacher during grade school, a middle school math teacher, a Catholic Priest, a high school biology teacher, and several professors in medical school.

I remember vividly the childhood story of the famous German mathematician, Johann Carl Friedrich Gauss (1777–1855). According to my 7th and 8th grade math teacher, the young Gauss and his classmates were left alone in their one‐classroom school. The assignment during the teacher's absence was to add up all numbers from 1 to 100 (i.e., 1+2+3+4+5+……+100). When Gauss came up with the answer in an unexpectedly short time, his teacher accused him of cheating. My teacher presented us with the same problem. The praise that I received after having come up with a way toward solving this problem was enough for me to enjoy math even more than before and to want to become a mathematician.

I remember equally vividly a different situation when I was a junior faculty. During a particularly challenging time, a senior colleague was willing to listen and help me with a difficult decision that had a major impact on my professional career. Mentors along the way have made a lasting impression on me and on my career. Sometimes the significant influence was simply a short conversation after a presentation, while other times long‐term professional relationships were made such as those with my thesis advisor during medical school, clinical attendings during my pediatric residency and medical genetics fellowship training, two postdoctoral advisors, and senior faculty colleagues.

## Mentors in Literature and History

There is a Japanese expression “hearing a mentor's coughing”. It means that a student can learn a lot by just being with his/her mentor in person even if there is no or little verbal communication (this saying was provided by Yoichi Matsubara). Literature provides many examples of mentors, usually in a setting where an older, more experienced person takes a younger, less experienced person under his/her wings. In his 1931 novel (published in 1943) “The Glass Bead Game” (*Das Glasperlenspiel*), which is set several centuries in the future, Herman Hesse details a number of teacher–student relationships. The main protagonist, Joseph Knecht, is both a mentee and eventually a mentor to several who are under his care. In Umberto Eco's 1986 novel: “The Name of the Rose” (*Il nome della rosa*), set in 1327 Northern Italy, an older Franciscan monk, William of Baskerville, teaches by example his travel companion, the Benedictine novice Adso of Melk. Examples from 20th and 21st century pop culture include Obi‐Wan Kenobi and Luke Skywalker in *Star Wars*, and, of course Dumbledore and Harry Potter in the *Harry Potter* series.

Mentoring in history comes in different flavors. The passing down of knowledge from one generation to the next has been practiced for millennia and is known as the concept of guru‐parampara or disciplic succession. However, most historical examples include specific mentor/mentee pairs such as Socrates and Plato, Ralph Waldo Emerson and Henry David Thoreau, Paul Gauguin and Vincent Van Gogh, William Osler and Harvey Cushing, G.H. Hardy and Srinivasa Ramanujan, Annie Sullivan and Helen Keller, Richard Wright and Ralph Ellison, Robert Gorlin and M. Michael Cohen, Jr., Barbara Walters and Oprah Winfrey, Billie Jean King and Chris Evert, Larry Summers and Sheryl Sandberg, and many more.

## Modern Mentoring in Medicine and Science

Inspiring and guiding the next generation is influenced by many factors, including the mentor and the setting. As an academic physician‐scientist, Nobel Laureate Robert Lefkowitz considers, “one of the most important things we do is mentor young trainee‐scientists” (Lefkowitz 2015). He lists key ingredients of a successful mentor‐mentee relationship: providing a strong role model, leading by example, sharing a sense of wonder and curiosity, spreading enthusiasm, a passion for knowledge, and “liberal doses of humor to encourage out of the box thinking” (Lefkowitz 2015).

Sambunjak et al. (2006, 2009) present a comprehensive overview and review of research of the “meaning and characteristics of mentoring in academic medicine.” They summarize desired qualities and actions of good mentors, barriers to mentoring and dysfunctional mentoring, and lastly strategies to improve mentoring. Characteristics of good mentors have been divided into three categories: personal, relational, and professional. Personal characteristics of a good mentor include: being altruistic, understanding, patient, honest, responsive, trustworthy, nonjudgmental, reliable, an active listener, and a motivator. Relational mentor qualities are: being accessible, dedicated to the mentee's best interest, able to identify potential strength, “a good match” in terms of practice style, vision and personality. A professional mentor is senior, well‐respected in his/her field, knowledgeable and experienced (summarized in Sambunjak et al. 2009). The authors conclude: “successful mentoring requires commitment and interpersonal skills of the mentor and mentee, but also a facilitating environment at academic medicine's institutions.” Levinson et al. (1991) reported on the specific challenges for women to identify female mentors and/or role models in leadership positions in academic medicine.

In addition to individual physicians or scientists who mentor, there are many institutionalized mentorship programs, like the Summer Internship Program in Biomedical Research (SIP) at the National Institutes of Health (https://www.training.nih.gov/programs/sip), prestigious internships in large companies like Ernest and Young (http://www.exceptionaley.com), Google (https://www.google.com/about/careers/students/), Apple (https://www.apple.com/jobs/us/students.html); Epic (https://careers.epic.com/Home/ViewPosition?id=270), Facebook (https://www.facebook.com/careers/university/internships/engineering), and many others.

## Mentoring in Medical Genetics and Genomic Medicine

For over 25 years, I have trained undergraduate, graduate students, and postdoctoral fellows in my human and medical genetics and genomics research laboratory. As the Director of the NIH Medical Genetics and Genomic Medicine Residency and Fellowship Programs, I have supervised and mentored over 150 physicians in clinical genetics and post‐doctoral fellows in the genetic laboratory specialties of cytogenetics, biochemical genetics, and molecular genetics. Despite having read numerous books on the art of mentoring and participating in workshops to further my skills, it is clear that my one‐on‐one encounters with my trainees have taught me the most about the process.

When discussing my experience with my fellow program directors, there are common themes throughout all of our practice as mentors. Mentoring is as individual as the respective mentor and mentee. Leading by example can be very influential. Most trainees in our programs are advanced in their training, highly motivated, and need little supervision and guidance. However, we found that regular interactions through weekly clinic conferences or lab meetings and the giving of immediate feedback to trainee presentations or resident‐patient interactions can be instrumental. More formal one‐on‐one meetings between trainee and mentor including documentation of previous goals and progress as required by the American Board of Medical Genetics and Genomics (ABMGG) have shown to be beneficial for both mentor and mentee.

Mentoring requires dedication and concerted effort to periodically and honestly review progress made by the trainee, obstacles encountered and assessment of performance in order to adjust and align goals with developing knowledge and skills. This process can be rewarding, time‐consuming and exhausting especially when performance, personal issues or even personalities impede progress. On occasion, there can be an urgency to mentoring and the need to be more directive and to help out at moment's notice in a challenging situation. At other times, the mentee may benefit from the experienced mentor who leads by example in taking the high road, by having a sounding board, and/or by injecting some sense of reality into a difficult situation. Ideally, the mentee should sense an environment of trust and sincerity in order for the most challenging issues to be unveiled and explored. However, in practice, the desired mentor‐mentee relationship is not always achievable. In cases where the match may not be ideal, efforts should be made to help the trainee identify other mentors who can establish a relationship that allows exploration of challenging issues, self‐limiting habits or behaviors that threaten the trainee's professional development.

## Proposal of Mentoring Through Mentors Without Borders

Ideally, every trainee has at his/her institution one primary, long‐term mentor, for example, the thesis advisor, the head of the laboratory, the director of the residency or fellowship program, or others. There may be additional secondary mentors, like the members of a thesis committee, clinical attendings, an assigned mentoring committee for tenure track faculty, etc. Lastly, trainees may seek yet additional advice from other students or faculty from the same institution who are outside their own training program. This system can work well at colleges, universities and other institutions of higher learning that are large enough to have a graduate or medical/dental school, several residency and fellowship programs, but less well at institutions with a small number of faculty and/or faculty with limited expertise relevant to a particular trainee. In addition to the potential lack of mentors at one's home institution, trainees may want to have different mentors for different aspects of their professional career. Lastly, every trainee is unique and may benefit from a mentor that works well with a particular trainee.

The concept of mentoring from afar is neither new nor simply theoretical, and has been practiced in different settings even without experiencing direct one‐to‐one mentoring on an ongoing basis. I am aware of a number of senior human and medical geneticists who have mentored trainees by phone, email, or occasional planned in‐person meetings at the annual conferences of professional societies, such as the American Society of Human Genetics, the European Society of Human Genetics, or the Japanese Society of Human Genetics. Furthermore, E‐mentoring is an actual term for mentoring through the use of online software and/or email. Mentoring databases exist, for example, one for medical students on how to identify a mentor from the American College of Physicians (ACP) (https://www.acponline.org/membership/medical-students/find-a-mentor).

We propose an international mentoring network that allows trainee geneticists to identify mentors from a list of volunteers who are not at one's own institution. *Mentors without Borders* (www.MentorsWithoutBorders.com) is an experiment, a matchmaker between a junior and a senior professional. These mentors do not replace the mentors at the home institution but allow the mentee, if desired, to identify mentors outside of their own institution. We envision that different ways of communicating and/or different mentor‐mentee relationships may prove beneficial to the trainee and the mentor. Similar to the ACP website for medical students (above), the *Mentors without Borders* website will start with a *Find a Mentor* section. Other features may include a list server to allow networking and email exchanges between trainees and trainees and mentors and possibly online lectures and/training modules on issues important to a wide of trainees.

## Conclusion

Genetics and genomics continues to be a rapidly evolving area. The changes and the potential in this area of medicine and science, while exciting, can also be overwhelming. It will also take hard work to make sure that new techniques and discoveries can spread to benefit many, rather than being concentrated in a few select institutions. We sincerely hope that our program can contribute to the field by investing and believing in the next generation of mentors.

## Helpful Links

These are guidelines for mentors, mentees, and institutions to foster the development of good mentors:

- *Nature*'*s* guide: http://www.nature.com/nature/journal/v447/n7146/full/447791a.html
- US National Academy of Sciences: http://www.nap.edu/read/5789/chapter/1
- European Commission report on best practices in mentoring: http://ec.europa.eu/research/science-society/document_library/pdf_06/technopolis-exec-sum_en.pdf
- NIH guide for choosing a mentor: https://www.training.nih.gov/mentoring_guidelines
- Recommendations from the American Association for the Advancement of Science (AAAS) for academic institutions: http://ehrweb.aaas.org/sciMentoring/Recommendations.pdf

## Conflict of Interest

None declared.
